# Low-Cost *Ka*-Band Satellite Receiver Data Preprocessing for Tropospheric Propagation Studies

**DOI:** 10.3390/s22031043

**Published:** 2022-01-28

**Authors:** Vicente Pastoriza-Santos, Fernando Machado, Dalia Nandi, Fernando Pérez-Fontán

**Affiliations:** 1 atlanTTic Research Center for Telecommunications Technologies, Universidade de Vigo, 36310 Vigo, Spain; fmachado@uvigo.es (F.M.); fpfontan@tsc.uvigo.es (F.P.-F.); 2 Indian Institute of Information Technology Kalyani, Kalyani 741235, West Bengal, India; dalia@iiitkalyani.ac.in

**Keywords:** beacon receiver, *Ka*-band, measurement campaign, rain attenuation, satellite measurements, template

## Abstract

Satellite tropospheric propagation studies strongly rely on beacon receiver measurements. We were interested in performing a measurement campaign to characterize rain attenuation statistics. In this article, we outline some of the characteristics and drawbacks one faces when trying to perform a radio wave satellite beacon propagation experiment at the *Ka*-band with low-cost measurement equipment. We used an affordable beacon receiver consisting of a commercial low-noise block down-converter, an outdoor dual-reflector antenna, and a software-defined radio unit. To measure rain attenuation events, we needed to work out where the reference signal level was at all times. However, as we did not have a radiometer to remove the impact of gases and clouds, since it is a very expensive device, we used a procedure that involved the subtraction of a stable and reliable reference level (template) from the raw received beacon level. This template was extracted from observations during non-rainy periods. The procedure implemented for extracting the template was based on the same data processing methodology used by other authors in this field. Here, we describe through specific examples the main characteristics of the templates extracted on non-rainy days, as well as the impact of some meteorological parameters and unavoidable, but small antenna pointing errors.

## 1. Introduction

A measurement campaign recording the *Ka*-band beacon transmitted by the KaSAT satellite is being carried out at the University of Vigo campus in Vigo (Spain). This location, on the NW coast of Spain, corresponds to a temperate, oceanic climate. The beacon receiver is a simple, affordable one with a commercial Low-Noise Block down-converter (LNB), an outdoor dual reflector antenna fitted with a radome, and a Software-Defined Radio (SDR) unit, performing further signal down-conversion and discretization to pass to a Personal Computer (PC). Additionally, meteorological information is obtained from a co-located weather station. Moreover, as the receiver is sited close to Vigo Airport, cloudiness data are also available via METeorological Aerodrome Report (METAR) files.

This experiment complements another on-going one (running since 2015) at *Ka*- and *Q*-band measuring the beacons transmitted by AlphaSAT, as part of an Italian Space Agency/European Space Agency (ASI/ESA) experiment [1,2,3]. Due to the azimuth separation between KaSAT and AlphaSAT in Vigo, approximately $19^{\circ }$, we are also gathering orbit diversity information.

Note that carrying out measurements with a commercial satellite’s beacon (KaSAT), we may be subjected to problems not present in the case of specialized, highly stable payloads, as is the case of AlphaSAT [2]. In commercial satellites, we are not in control of the actual transmitted power, which may change due to operational requirements. The only thing that can be done is to try and detect these events and remove them by calculating and extracting a template (see below). Similarly, the use of a commercial LNB can also introduce variations in the received signal power due to thermally induced changes in the electronics. Our LNB (see Section 2.1.1 and [4]) is subjected to gain variations due to the ambient temperature. Its maximum operational range is $-40{\;}^{\circ }$C to $+60{\;}^{\circ }$C, and the gain changes can be of the order of 5 dB. At our location during summertime (worst case), the maximum temperature range is of the order of $12{\;}^{\circ }$C with an estimated gain variation of under 2 dB (private communication with the manufacturer). This must be compensated for by subtracting the template.

One further problem arises due to the fact that the satellite experiences very small position oscillations of no more than $\pm 0.1^{\circ }$ (stationkeeping box) [5,6]. This, combined with the presence of possible small pointing errors—in this experiment, we used fixed pointing—can lead to unwanted signal variations. Again, these must be first minimized by a good pointing and, then, any residual errors induced removed, using the template. However, given as we used a fairly small receive antenna of 65 cm diameter, this is equivalent to approximately a beamwidth of $1.6^{\circ }$, much wider than the satellite’s stationkeeping box. This means that pointing errors will be a second-order factor in comparison with the temperature-induced LNB gain changes.

We were interested in characterizing rain attenuation statistics. However, in addition to rain, the received beacon signal power level can also be affected by other phenomena: gases, clouds, scintillation [7], temperature fluctuations of the unit electronics, antenna strut thermal expansion [8], the accuracy of the satellite tracking [5], etc.

The raw received beacon signal in dBm can be calculated as [5,9,10]:

(1) $$P_{r}=EIRP-L_{fs}+G_{ra}+G_{rx}-A_{g}-A_{c}-A_{r}-A_{s},$$

where *EIRP* is the satellite’s Equivalent Isotropically Radiated Power (dBm), $L_{fs}$ is the free space loss (dB), $G_{ra}$ is the receiving antenna gain (dBi), and $G_{rx}$ is the receiver gain (dB). The last four terms in (1) correspond to different propagation effects and are expressed in dB: $A_{g}$ includes water vapor and oxygen loss; $A_{c}$ corresponds to cloud loss; $A_{r}$ is the rain-induced attenuation; $A_{s}$ embodies the scintillation. (Sometimes, the scintillation is represented as a gain, and thus, the sign in the above equation is changed to +.)

The total tropospheric attenuation *A* (dB) is the sum of these four terms [5,9,10]:

(2) $$A=A_{g}+A_{c}+A_{r}+A_{s}.$$

As said, we were interested in measuring rain attenuation, $A_{r}$. One first option to remove the impact of gases and clouds is by measuring them directly using a radiometer, as in [11,12,13]. This is a sophisticated piece of hardware, even more expensive than a beacon receiver, and was not available to us.

Radiometer data could be replaced by alternative data sources. Thus, Global Navigation Satellite System (GNSS)-derived measurements provide an excellent means of predicting water vapor attenuation, hence helping define the reference level [13]. Another option would be using numerical weather data (prediction/reanalysis) particularized for the tropospheric volume around the Earth station [14]. The latter technique is quite effective in determining water vapor attenuation [15,16], even in tropical areas [17]; however, the $A_{c}$ estimation is not so reliable, at least on an event-by-event basis.

In our case, faced with the impossibility of using a radiometer to remove cloud and gas attenuation directly [12,13], we relied on the fact that the attenuation variations caused by rain are much faster than those due to, on the one hand, the combined effect of a likely small mispointing of the antenna together with the movement of the satellite and, on the other, to gases, clouds, and the daily thermal cycle on the LNB electronics.

We can subject the acquired time series to a high-pass filtering process to remove these effects, thus retaining the faster ones, namely $A_{r}$ and $A_{s}$. The specific procedure involves subtracting a stable, reliable set of reference levels (template) from the raw received beacon levels. The template is mostly extracted from observations during non-rainy periods. Note that this operation removes all slowly varying effects. The result of this procedure is the excess attenuation that contains $A_{r}$ and $A_{s}$. The excess attenuation is the variation of the raw received beacon signal with respect to the template.

Although it has not been installed in our system, it is interesting to note that thermal effects on the unit electronics can be partially compensated for by using Peltier cells attached to the LNB metal enclosure, as in [18]. A good approach to using this technique is to keep the LNB at a constant temperature of, for example, $50{\;}^{\circ }$C. In the referenced case, this was easier because their receiver was totally located indoors looking at AlphaSAT through a millimeter wave transparent window, while our Radio-Frequency (RF) unit was outdoors (Section 2.1.1).

Moreover, looking into the work reported by others, in [8], the authors discussed that the level fluctuations they observed were due to antenna strut thermal expansion. Their setup (4.2 m dish, ≈56.5 dBi gain, ≈$0.25^{\circ }$ beamwidth) included a very large dish and a temperature-stable receiver. However, our antenna (65 cm dish, ≈40.5 dBi gain, ≈$1.6^{\circ }$ beamwidth) was significantly smaller and the radiation pattern sufficiently wide so that thermal, mechanical expansion was not an issue.

Finally, to separate $A_{r}$ from $A_{s}$ in the excess attenuation, low-pass filtering is often used to remove the fast-varying attenuation due to scintillation, which has spectral components up to about 3 Hz, while rain is much slower [19]. Typical cut-off frequencies to remove $A_{s}$ are on the order of 0.02–0.04 Hz [19,20]. This is normally done with a running mean filter with a duration of between 1 min and 2 min [10,21]. Sometimes, more sophisticated filters, e.g., a Butterworth of fifth order, as in [19], or a squared-cosine, as in [22,23,24], can be used.

In this paper, we concentrated on the preprocessing phase indicating “conditioning” of the row data. Further, actual identification of rain events and extraction of monthly, seasonal, or yearly statistics of first order (CDFs, cumulative distributions) or second order (time dependent) such as event durations, slopes, etc., need to be carried out, but are outside of the scope of this paper. For a detailed description of these tasks, refer, for example, to [2,3].

This paper is organized as follows. Section 2.1 presents the experimental setup, including the beacon receiver and the available local meteorological data. The template extraction methodology is described in Section 2.2. Section 3 shows several templates extracted on non-rainy days. We give several correlation analyses between templates and meteorological parameters. We tried to find explanations for the relevant characteristics noticed on the studied time series. Finally, Section 4 draws some conclusions.

## 2. Materials and Methods

### 2.1. Experimental Setup

At the University of Vigo campus in Vigo, Spain, with coordinates $42.170^{\circ }$ N, $8.688^{\circ }$ W and 447 m a.m.s.l., a *Ka*-band beacon receiver pointing toward KaSAT, $9^{\circ }$ E, was set up. This experiment has been running from the beginning of 2018.

The receiver was tuned to the 19.68 GHz beacon. The geometry of the link is as follows: azimuth $154.6^{\circ }$, elevation approximately $38.1^{\circ }$, and linear polarization with an angle with respect to the local horizontal axes, τ, of $18.5^{\circ }$. The receive antenna is a dual-reflector parabolic dish. The measurement bandwidth is approximately 50 Hz with a data rate of over 10 Hz. The measurement dynamic range exceeds 20 dB.

Our setup also includes a meteorological station co-located with the beacon receiver that measures the following variables: rain rate derived from two optical disdrometers [25] and three standard tipping-bucket rain gauges [26], wind direction and speed, relative humidity, ambient temperature, and atmospheric pressure from their corresponding sensors (all with a 1 min time resolution). Furthermore, from the METAR files provided by the OGIMET service [27], it is possible to access the meteorological data gathered at Vigo Airport, less than 10 km from the beacon receiver, with a half-hour time resolution: mainly the cloud cover (in oktas) and cloud heights (in hundreds of feet).

The beacon receiver was built using off-the-shelf elements, which were grouped into two main parts, as follows.

#### 2.1.1. The RF Outdoor Unit

The outdoor unit comprises a 65 cm dual-reflector parabolic dish manufactured by ALCOMA (Model UNI2-18) [28]. Although this antenna is designed for terrestrial point-to-point links, small modifications allowed us to use it for any satellite link with linear polarization with any orientation, not only vertical or horizontal, as in the terrestrial case. The second element is an LNB from Norsat (Model BDC-9000XBN) [4] that has its own, fairly stable local oscillator. The LNB output connector (N-type 50 Ω) is hooked up to the indoor unit by means of a 3 m low-loss coaxial cable (0.5 dB/m). The Intermediate-Frequency (IF) cable provides 15 V DC power to the LNB.

The antenna provides a gain of 39 dBi at mid-band (11.70–19.70 GHz), and its input port is a rectangular waveguide (WR42). The LNB covers the frequency range 19.20–20.20 GHz, providing low-noise amplification and block conversion to the standard 0.950–1.950 GHz satellite receiver IF. This unit has an overall gain of 55 dB and a noise figure of 1.3 dB. We believe that this piece of equipment, being a commercial, “mass”-market one, is the main source of diurnal variation observed in the template and is due to its exposure to changes in ambient temperature (Section 3).

#### 2.1.2. The Indoor Unit

This takes care of a second frequency conversion, signal processing, and data logging. It is composed of an SDR unit (bladeRF x40) [29] connected to a PC. The PC runs under Windows 10 and carries out both signal processing and data logging tasks.

The bladeRF SDR is fit with an RF transceiver, which performs a second down-conversion and quadrature sampling (up to 40 Msps outputting 12 bit *IQ* samples). In spite of the availability of this large bandwidth, we set the SDR unit to its minimum tunable bandwidth, i.e., 1.5 MHz, to be centered approximately about the beacon frequency, and to a sampling rate of 2 Msps.

To avoid unexpected problems, the received beacon signal was moved away from the DC frequency and located somewhere midway between 0 and one-half of $f_{s}$ (sampling rate). It is common in affordable SDR hardware to observe a significant DC component at a complex baseband, thus the decision of tuning the beacon away from 0 Hz for measuring its power. Since no further filtering is performed at this point, the baseband presents flat spectral characteristics except for samples very close to the selected spectrum edges. Furthermore, the beacon (19,680 MHz) is situated in a quiet part of the transponder’s bandwidth, some 20 MHz away from the limit of the modulated signal spectrum (19,700 MHz). Note also that our measurement bandwidth, as said above, is only 1.5 MHz, which allows achieving the wanted spectral flatness. The closest spot beam center frequency is 19,818.75 MHz [30]. The obtained *I* and *Q* components are passed to the PC via a USB 3.0 port. Finally, these signals are processed and stored along with the resulting measurements in the PC. Signal processing involving the identification and measurement of the beacon at the baseband is performed.

The signal processing is very simple: it involves computing a long Fast Fourier Transform (FFT) of consecutive, non-overlapping signal blocks, identification of the beacon signal at the baseband, and power summing three spectral FFT bins around the beacon signal [31]. A single spectral sample is not enough unless the sample is exactly at the center of the frequency bin. Given the presence of drift, it is simpler to power sum the peak and side frequency bins. We chose an FFT of size $2^{18}$ points, which together with a sampling rate of 2 MHz yielded a spectral bin size of 7.6294 Hz and also an output data rate by the same amount. Note that this final sampling rate fulfills the Nyquist criterion for the fastest of the various phenomena involved, i.e., scintillation with negligible spectral components beyond 3 Hz. We chose to measure the power in three adjacent bins, one at each side of that with the spectral maximum. This is equivalent to a measurement bandwidth of ≈23 Hz. Non-overlapping FFTs were employed in order not to excessively burden the controlling computer so that real-time measurements could be obtained.

The resulting information represents the variations in the power level of the received beacon signal, *S*, plus noise, *N*. Note that the noise originates both at the receiver (noise factor) and the antenna (antenna noise temperature). Thus, we are actually measuring S+N instead of only *S*. With computer simulations assuming Gaussian *I* and *Q* noise components plus a steady direct signal, we verified that as long as we had over about 10 dB Signal-power-to-Noise-power Ratio (v), we could obtain fairly uncorrupted signal samples.

Many setups, e.g., [18,32], including ours, also perform an additional calculation of the overall power at the baseband (from $-f_{s}/2$ to $f_{s}/2$). The purpose of this is to help identify rain events. This is similar to what radiometers do, albeit with much more stable, constant gain receivers. In our case, we cannot tell an increase in water vapor or the presence of clouds through this calculation, but still, we can obtain a clear indication of the presence of rain events: a decrease in the beacon power is accompanied by an increase in the noise.

**Figure 1:**
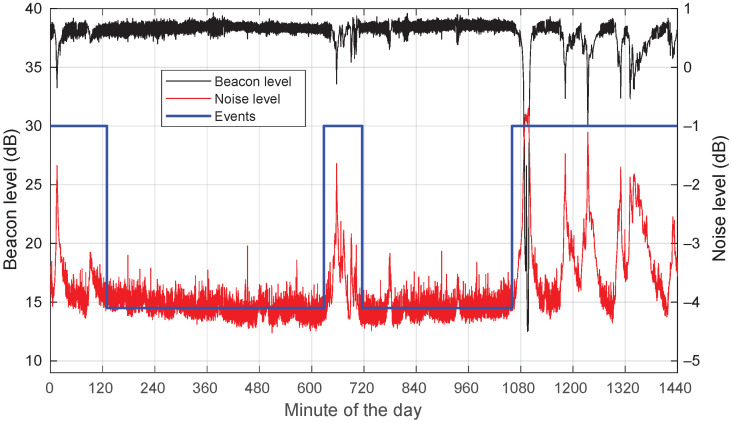
Example of KaSAT beacon power level and received noise level for a day with three rain events: 24 April 2021. The identified rain events are also illustrated. Note the increase in noise temperature when it rains together with a drop in the received beacon power.

As an example, Figure 1 shows the measured power level of the KaSAT beacon and background noise for a day with three rain events. It is clear how the noise power increase marks the time limits of the rain events. Note that the signal levels in the ordinates do not correspond to actual power values in dBm; they are the numerical result of applying an FFT to the *I* and *Q* samples out of the analog-to-digital converter. In the series, the relative values between time samples have a physical meaning, but their absolute values are meaningless.

**Figure 2:**
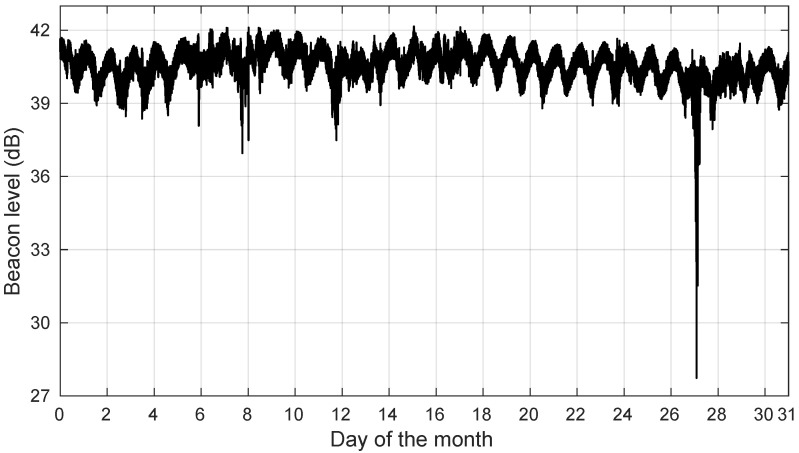
Example of the raw beacon level for the whole of August 2018. A mainly dry month is depicted except for a few rain events. Note the daily signal oscillations, mostly caused by changes in the outdoor unit’s gain (thermal effects) and, to a lesser degree, to satellite orbit variations.

In Figure 2, we illustrate the measured power level of the KaSAT beacon for the whole month of August 2018. Note in Figure 2 how the raw beacon level undergoes a daily cycle on days with a mostly clear sky.

### 2.2. Extraction of the Daily Template

As mentioned in Section 1, given the characteristics of our measurement system, to estimate the excess attenuation, we subjected the time series of the acquired beacon level to a filtering process consisting of subtracting a template from the time series. The procedure implemented for extracting the template was based on the data processing methodology described in [33], which is similar to those found in [20,22,34].

Roughly, the procedure to compute a daily template consisted of the following steps:

1. Three days of data selection: time synchronized beacon and meteorological data of the previous, current, and next days;
2. Outlier detection based on the standard deviation of the mean value of the raw data: These mean values were computed using a 2 min moving average filter. When the difference between the beacon level and its moving average is larger than the standard deviation multiplied by a coverage factor, then the sample is recognized as an outlier and flagged as invalid data. In this work, a coverage factor of 5 was used;
3. Detection of abnormal amplitude changes (exceeding a defined threshold) from one sample to another: In this study, the samples exceeding a threshold of 1 were considered as spikes and flagged as invalid data;
4. Identification of rainy periods: Data corresponding to rain events are flagged as invalid data;
5. A repair operation: Missing and invalid data are replaced by linear interpolation from the valid data;
6. After having inspected and repaired the selected data, template extraction itself is performed. To identify the template, we performed a low-pass filter processing that should be able to follow the fairly slow changes in water vapor in the atmosphere, with time constants of hours, and the presence of clouds, with time constants faster, but still slower than rain. In addition, we had to follow both the combined effect of receive antenna mispointing plus satellite orbit perturbation and the LNB gain oscillations due to the thermal variations, which clearly have a full one-day duration. The template was computed with an FFT by considering only those components whose period was longer than 8 min. Several tests were carried out, and this seemed to be the optimum value in our case.

**Figure 3:**
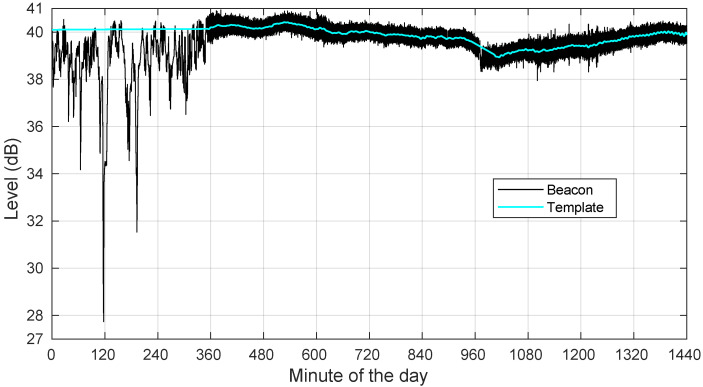
Example of raw beacon level and extracted template for a rainy day: 28 August 2018.

To take an example, Figure 3 shows the raw received beacon level (our row data) during a rainy day (28 August 2018). In Figure 3, the daily pattern is visible in the second half of the plot. The difficulty arises during the rain episode in the early hours. During the rainy period, the extraction of the template from raw data was not possible, and rainy data were replaced (Steps 4 and 5 of the template extraction procedure).

**Figure 4:**
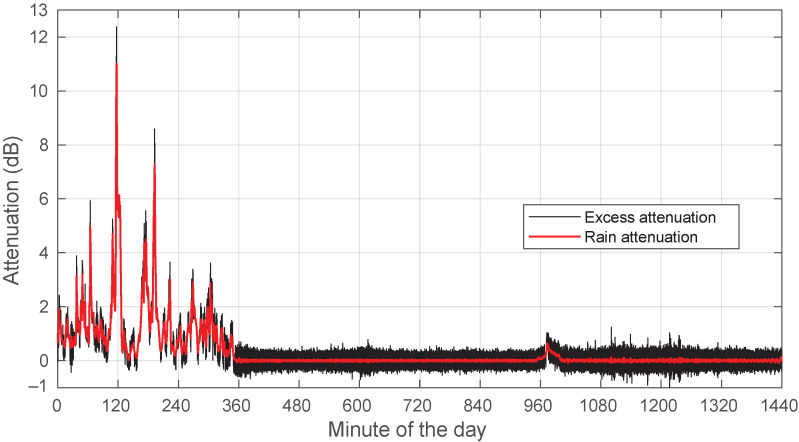
Example of excess and rain attenuation for a rainy day: 28 August 2018.

As in Figure 1 and Figure 2, the signal levels in Figure 3 do not correspond to actual power values in dBm. It is only after the template has been identified and subtracted from the raw data that the obtained levels have any meaning: they are now relative levels with respect to the set reference level. The resulting time series is the excess attenuation. Finally, to separate $A_{r}$ from $A_{s}$ in the excess attenuation, we applied a squared-cosine window low-pass filter with a duration of 28.76 s. Figure 4 shows the excess attenuation and $A_{r}$ for 28 August 2018.

## 3. Results

**Figure 5:**
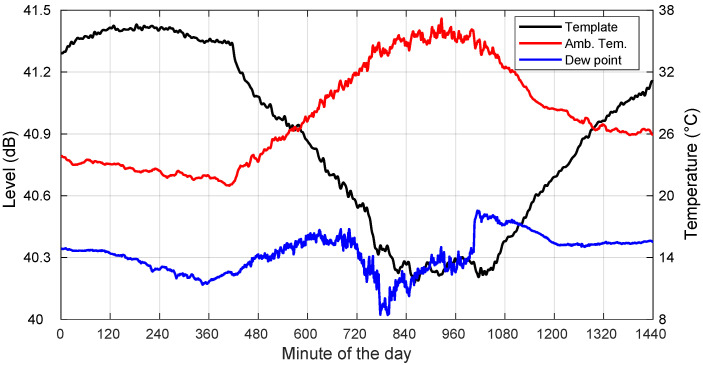
Template, ambient temperature, and dew point for a clear-sky day: 19 August 2018.

In this section, we go on to describe, through specific examples, the main characteristics of the templates extracted on non-rainy days, as well as the impact of some local meteorological parameters. Note that our ensuing analyses were based on locally measured meteorological information at the receiver location. However, it is worth mentioning that it is the whole path’s parameters that condition the measured received levels. In Figure 5, we analyze a clear-sky day (19 August 2018) recording together with associated concurrent local meteorological information. We show the extracted template, the ambient temperature, and the dew point along the day. Note again that the absolute signal levels shown here were not calibrated to dBm and were the result of the processing of the in-phase and quadrature digitized input signal.

Since the overall raw data are also affected by some level of unavoidable mispointing, our correlation analyses should be regarded with care given that we only studied here the impact of the ambient temperature and other meteorological variables. Even though we are aware of the fact that the correlation levels observed for meteorological parameters can be disturbed by the correlation between the raw data and the pointing errors, these results continue to be relevant for deriving that particular day’s template. The fact that both the thermal cycles and the satellite positioning errors show the same period does not completely imply that the correlation coefficients due to one phenomenon and the other have the same strength.

We saw a clear negative correlation with the ambient temperature. Furthermore, this temperature was always far from the dew point. This kind of behavior has been observed in most of our non-rainy recordings, with high levels of anti-correlation always below −0.9.

**Figure 6:**
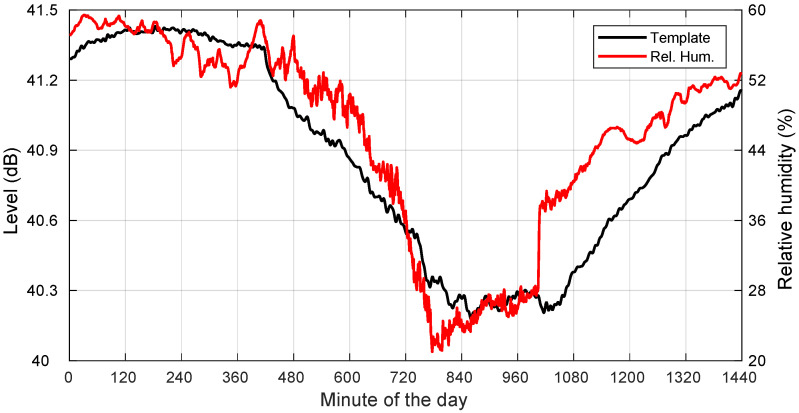
Template and relative humidity for a clear-sky day: 19 August 2018.

Additionally, we also include in Figure 6 a plot showing the template and the relative humidity, where high correlation values are also apparent, indicating another possible relevant influencing factor in the evolution of the template.

**Figure 7:**
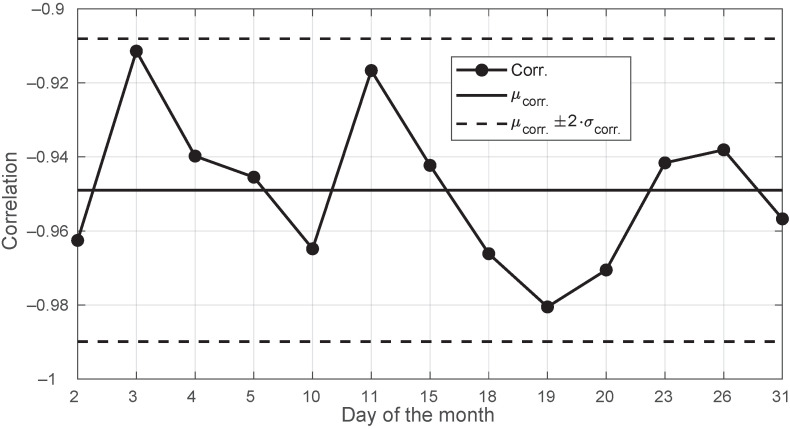
Correlations between template and ambient temperature for the days in August 2018 with all full-day clear sky. Days: 2–5, 10–11, 15, 18–20, 23, 26, and 31.

Clear sky is a very common condition during the year. The negative correlation between template and temperature is significant. For the month of August 2018, full-day (no morning fog) clear-sky conditions were met during Days 2–5, 10–11, 15, 18–20, 23, 26, and 31. Their corresponding correlation values with the ambient temperature are listed in Figure 7. They never went above −0.91.

**Figure 8:**
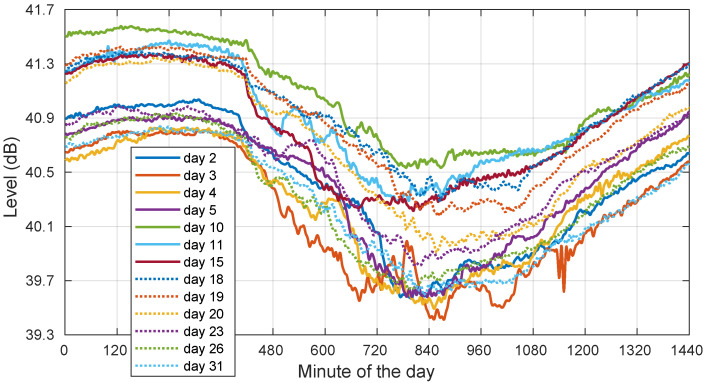
Templates for the days in August 2018 with all full-day clear sky. Days: 2–5, 10–11, 15, 18–20, 23, 26, and 31.

**Figure 9:**
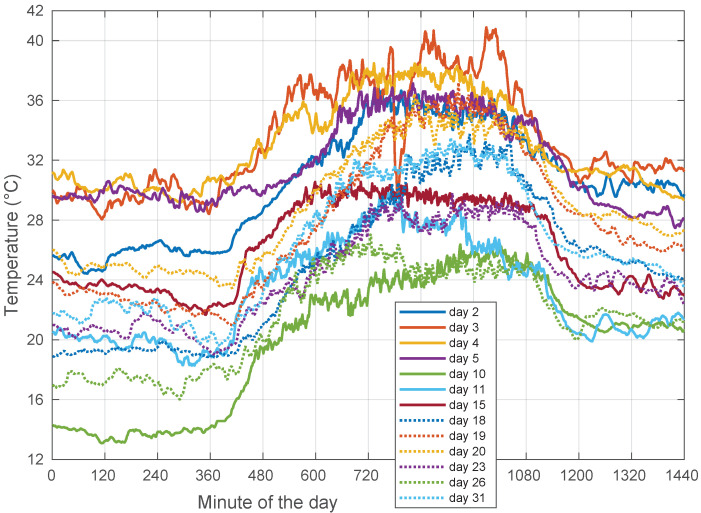
Ambient temperature for the days in August 2018 with all full-day clear sky. Days: 2–5, 10–11, 15, 18–20, 23, 26, and 31.

However, we did not find a deterministic behavior in the observed signal levels, as illustrated in Figure 8, which shows that the templates extracted in clear-sky days were not necessarily identical from day to day. As complementary information, we also provide the corresponding day-to-day ambient temperature plots in Figure 9.

Coming back to the templates in Figure 8, the changes in their daily average levels, not in their peak-to-peak dynamic ranges, could be fundamentally attributed to a longer term upward or downward drift in the orbit cycle, which is only reset when stationkeeping maneuvers are carried out on a periodic basis.

These effects completely mask out the water vapor and cloud effects, which are of lower magnitude. Under these constraints, the use of an all-time, single template is not feasible, and thus, it is necessary to employ the current day’s template (or, at most, one involving the previous and following days.)

**Figure 10:**
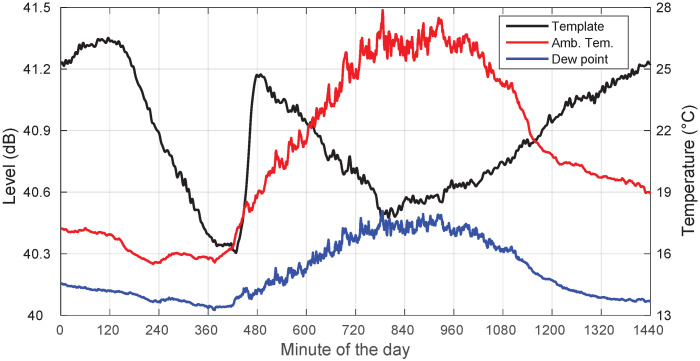
Template, ambient temperature, and dew point for a clear-sky day with possible condensation on the radome: 17 August 2018.

Now, we go on to present a case, also often observed, with possible condensation on the antenna’s radome taking place. Figure 10 shows the same plot as in Figure 5, now for 17 August 2018. Here, we observe a strange artifact in the extracted template with a drop of a few tens of one dB during the early hours of the day, before and during sunrise. This drop then disappears, and the normal daily cycle is resumed. In this case, the correlation with the ambient temperature was −0.41. We can see that this strange episode took place during a period of time when the ambient temperature was close to the dew point. We assumed that this effect was probably caused by condensation on the antenna’s radome.

**Figure 11:**
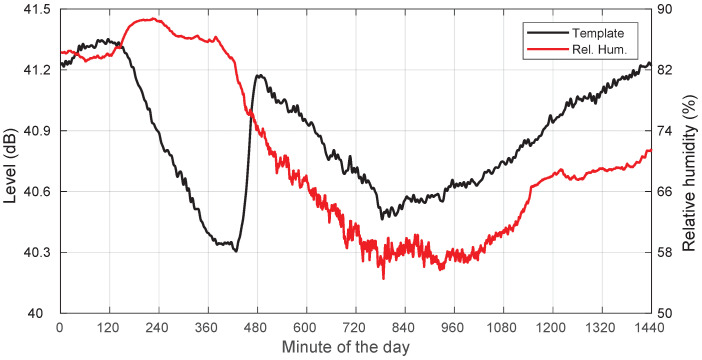
Template and relative humidity for a clear-sky day with possible condensation on the radome: 17 August 2018.

Furthermore, we have a look at the dependency between template and relative humidity (*RH*) in Figure 11. We can see that the level dip corresponds to a period with significant *RH*, close to 100%, consistent with the condensation hypothesis. In clear-sky days, as in Figure 6, the *RH* is usually much smaller.

**Figure 12:**
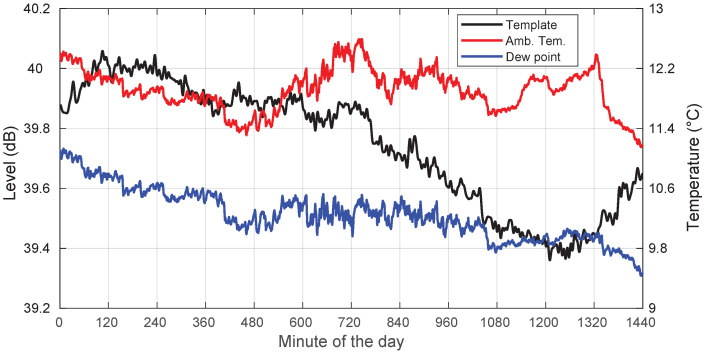
Template, ambient temperature, and dew point for a cloudy-sky day: 3 November 2018.

**Figure 13:**
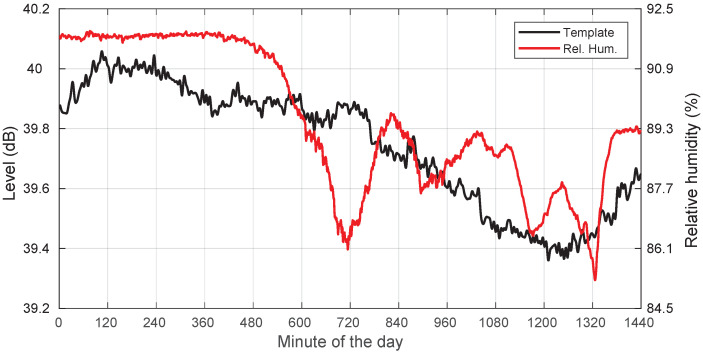
Template and relative humidity for a cloudy-sky day: 3 November 2018.

Finally, we looked at the recordings from 3 November 2018. This was a very cloudy day with no rain. Again, Figure 12 and Figure 13 illustrate the template and the concurrent local meteorological parameters. From the METAR files, it was obtained that the eight oktas of the sky were covered throughout the day (8/8 of sky completely overcast, no breaks or openings).

On this cloudy day, the template excursion was around 0.65 dB peak-to-peak, significantly smaller than on a sunny, warmer day in August (about 1.2 dB peak-to-peak; Figure 8.) The corresponding daily ambient temperature ranges were $1{\;}^{\circ }$C and at least $8{\;}^{\circ }$C, respectively. These differences can be attributed mostly to thermal effects on the LNB.

Now, coming back to the cloudy-day case, the relative humidity was also significant, and the ambient temperature was close to the dew point. In any event, it seemed that the average beacon level was slightly lower than in clear-sky days, which should mean that there is a constant additional attenuation due to water vapor and the clouds.

A final word is about the fact that we were always carrying out relative measurements of rain attenuation with respect to a daily template. This made our post-processed measurements insensitive to possible drops in the signal level for a few hours or days due to operational reasons. Provided that both ends of the rain event were affected by the same signal level, we relied on the fact that daily templates are capable of removing not only gas, cloud, thermal, and daily antenna mispointing effects, but also a drop in the satellite’s transmit power.

## 4. Conclusions

In this paper, we illustrated, with the aid of received signal and concurrent meteorological recordings, some of the difficulties one faces when trying to perform a millimeter wave satellite beacon propagation experiment lasting over periods of years when affordable measurement equipment is utilized.

The main challenge was determining a sequence of reference levels representing the 0 dB excess attenuation level (template). There is an underlying daily cycle in the received satellite signal, caused mainly by thermal effects on the outdoor unit electronics. Antenna mispointing can also play a significant role, but not in our case, since we used a large beamwidth antenna. The observed cycles are more remarkable when the temperature dynamic range is larger. It was shown that during cloudy days, the signal excursion is smaller and the daily average levels may be lower due to the presence of water vapor and cloud attenuation. We also observed how it is possible to detect water condensation on the radome, giving rise to deformations of the expected template.

## Data Availability

Not applicable.

## Abbreviations

The following abbreviations are used in this manuscript:

a.m.s.l.	above mean sea level
ASI	Agenzia Spaziale Italiana (Italian Space Agency)
CDF	Cumulative Distribution Function
EIRP	Equivalent Isotropically Radiated Power
ESA	European Space Agency
FFT	Fast Fourier Transform
GNSS	Global Navigation Satellite System
IF	Intermediate Frequency
LNB	Low-Noise Block
METAR	METeorological Aerodrome Report
N	Noise
PC	Personal Computer
RF	Radio Frequency
RH	Relative Humidity
S	Received Beacon Signal
SDR	Software-Defined Radio
SNR	Signal-power-to-Noise-power Ratio
USB	Universal Serial Bus
